# The efficacy and safety of SGLT2 inhibitors for adjunctive treatment of type 1 diabetes: a systematic review and meta-analysis

**DOI:** 10.1038/srep44128

**Published:** 2017-03-09

**Authors:** Jiao Chen, Fang Fan, J. Y. Wang, Yang Long, C. L. Gao, R. C. Stanton, Yong Xu

**Affiliations:** 1 Department of Endocrinology, Affiliated Hospital of Southwest Medical College, Luzhou, Sichuan 646000, China; 2 Joslin Diabetes Center, Boston, MA, USA.; 3 Beth Israel Deaconess Medical Center, Boston, MA, Boston, MA, USA.; 4 Harvard Medical School, Boston, MA, USA.

## Abstract

To assess the efficacy and safety of the SGLT-2 inhibitors as adjunct therapy to insulin in T1DM, clinical trials indexed in PubMed, Cochrane Library, EMbase from inception through April 5, 2016. A meta-analysis was conducted on trials of SGLT-2 inhibitors in patients with T1DM on insulin therapy using RevMan 5.3 software. Of the 371 articles identified, ten met eligibility criteria. Seven clinical trials including four randomized controlled trials and 581 patients were included. Compared with the control group, SGLT-2 inhibitors group had significantly reduced fasting plasma glucose by 0.69 mmol/L [1.32; 0.07], glycosylated hemoglobin A1C by 0.37% [0.54; 0.20], body weight by 2.54 kg [3.48; 1.60] and total daily insulin dose by 6.22 IU [8.04; 4.40]. The total incidence of adverse events (AEs), hypoglycemia, and genital and urinary infections were also similar to placebo, while an increased incidence of diabetic ketoacidosis (DKA) (n = 16) was seen in SGLT-2 inhibitors group. The present study demonstrates that SGLT-2 inhibitors are effective as adjunct therapy to insulin in T1DM, heralding improved glycemic control, reduced body weight and total daily insulin dose without an increase in total AEs, hypoglycemia, or genital and urinary infections. However, the risk of DKA should be carefully monitored in future clinical trials.

Diabetes mellitus (DM) is the seventh leading cause of mortality worldwide, with a continually increasing prevalence and incidence^1^. Globally, in 2015 the disease prevalence was 415 million adults, with an estimated 318 million people at risk for development of DM, thereby rendering a large burden of disease for the foreseeable future^2^. Type 1 diabetes (T1DM) accounts for less than 5% of the total DM cases worldwide, affecting approximately 22 million adults and 0.4 million children^3^. Insulin replacement therapy remains the mainstay of treatment for T1DM, and initial intensive diabetes therapy was associated with a modestly lower all-cause mortality rate when compared with conventional therapy^4^. However, there remains a varying degree of side effects of insulin therapy including weight gain and risk of hypoglycemia. As a result, insulin therapy may have grave consequences, including increased comorbidities with attempts at strict glycemic control, or alternatively non-compliance and resulting poor glycemic control.

In recent years, the use of adjunctive therapies to insulin—those that improve glucose metabolism and reduce insulin side effects—have become a popular topic of interest. A number of oral anti-diabetic agents have been tested in clinical trials as insulin adjuncts for management of T1DM, including thiazolidinediones (TZDs), biguanides, glucagon-like peptide 1 (GLP-1) analogs, alpha glucosidase inhibitors, dipeptidyl peptidase-4 (DPP-4) inhibitors and sodium glucose co-transport-2 (SGLT-2) inhibitors^5^,^6^,^7^,^8^,^9^,^10^. Currently, pramlintide, a peptide hormone analog, is the only insulin adjunct that is approved by the U.S. Food and Drug Administration (FDA) for T1DM^11^. However, the optimal adjunct therapy for T1DM remains a focus of many clinical trials and research efforts. The discovery of phlorizin, the first pharmacological SGLT-2 inhibitor^12^, paved the way for rapid development of similar anti-diabetic drugs. This drug and others in its class inhibit glucose and sodium absorption from renal tubules, thereby improving glycemic control for DM. The efficacy and safety of SGLT-2 inhibitors as a single or combination therapy for treatment of type 2 diabetes (T2DM) has been demonstrated in a number of studies^13^,^14^,^15^,^16^. To date, clinical outcomes seen with SGLT-2 inhibitors include reduced plasma glucose, weight loss, decreased blood pressure, and improved lipid profiles, all of which are welcome benefits in patients with DM.

This unique mechanism of action and strong efficacy of SGLT-2 inhibitors in the treatment of T2DM suggested potential benefit for treatment of T1DM, and this theory has been explored and validated recently in several animal experiments and clinical studies^17^,^18^,^19^,^20^,^21^,^22^,^23^,^24^,^25^,^26^,^27^,^28^. Thus, the aim of the present study was to identify and critically appraise clinical trials which used SGLT-2 inhibitors as adjunct therapy to insulin in T1DM. To this end we conducted a systematic review and meta-analysis of the identified trials, and herein discuss the efficacy and safety of this adjunctive therapy in T1DM, as well as provide scientific evidence for reasonable clinical use.

## Materials and Methods

### Data Sources and Searches

An extensive search for clinical trials in PubMed, EMbase, the Cochrane Library and CENTRRAI (from inception through April 5, 2016) for the terms ‘SGLT2 inhibitor’, ‘Sodium-glucose cotransporter 2 inhibitor’, ‘dapagliflozin’, ‘BMS-512148’, ‘canagliflozin’, ‘JNJ-28431754’, ‘empagliflozin’, ‘BI-10773’, ‘ASP-1941’, ‘ipragliflozin’, ‘tofogliflozin’, ‘remogliflozin’, ‘GSK 189075’, ‘LX4211’, and ‘sergliflozin’ was performed. The search strategy was adapted for each of the other databases, collected all clinical trials on humans and back into the references of including study.

### Study selection

Titles and abstracts of all retrieved studies were reviewed independently by two reviewers (J Chen and F Fan) to identify relevant studies. Any discrepancies were resolved by discussion, with involvement of a third reviewer (JY Wang) when necessary. Included clinical trials which compared SGLT-2 inhibitors versus placebo or baseline carried out in adults with T1DM (according to the American Diabetes Association classification^29^), namely both genders; age greater than 18 years; and no limitation of follow up length, sample size, race or nationality. Case reports, editorials, letters to the editors, trials enrolling non-diabetes, T2DM, or subjects younger than 18 years, and treatment with other oral anti-hyperglycemic agents were excluded. Specific outcomes extracted were: (a) fasting plasma glucose (FPG), (b) postprandial plasma glucose (PPG), (c) glycated hemoglobin A1C (HbA1c), (d) body weight, (e) total daily insulin dosage (TDI), (f) incidence of adverse events (AEs), (g) hypoglycemic events, and (h) urinary tract or genital infections. Other secondary outcomes included plasma lipids, blood pressure, and the incidence of diabetic ketoacidosis (DKA).

### Data extraction and quality assessment

All retrieved studies were imported into the reference management software, EndNote X7. Two researchers (Chen J and Fan F) evaluated the methodology of the included clinical trials independently according to the recommended tool in Cochrane handbook 5.1.0 (5.1.0, http://handbook.Cochrane.org/)^30^, and subsequently cross-checked these studies. Any resulting discrepancies were resolved by discussion or judged by a third reviewer when necessary. The quality of randomized controlled trials (RCTs) was assessed following recommendations as described in the Cochrane Handbook for Systematic Reviews of Interventions. These recommendations include randomization, blinding, incomplete outcome data, outcome reporting, and other bias. Non-randomized controlled trials (NCTs) were assessed using the the parameters proposed by MINORS, and labeled as “low bias risk”, “unknown bias risk” or “high bias risk” for notations of A, B, or C, respectively. The judgement was not used as criterion for the selection of trials, some items were used only for descriptive purpose.

### Data synthesis and analysis

All outcomes were pooled using RevMan5.3 software, which was provided by the Cochrane Collaboration. To integrate the principal outcomes according to the type and dose of SGLT-2 inhibitors following the recommendation of Cochrane handbook, data variables were converted into standard units. For continuous outcome variables (e.g., FPG) and dichotomous data (such as hypoglycemic events, AEs, etc.), differences were calculated by weighted mean differences (WMDs) and relative risk (RR). Heterogeneity was assessed using the I^2^ statistic, where I^2^ values of 25, 50, and 75% indicated low, medium, and high heterogeneity, respectively. In instances with I^2^ < 50%, the fixed-effect model with the Mantel-Haenszel method was used; while an I^2^ ≥ 50% is considered representative of statistical heterogeneity. To analyze the sources of heterogeneity, subgroup or sensitivity analyses was performed when necessary. The random effects model was also used to analyze the unknown reason of heterogeneity, and the presence of publication-related bias was evaluated via funnel plots.

## Results

### Retrieved studies

**Figure 1:**
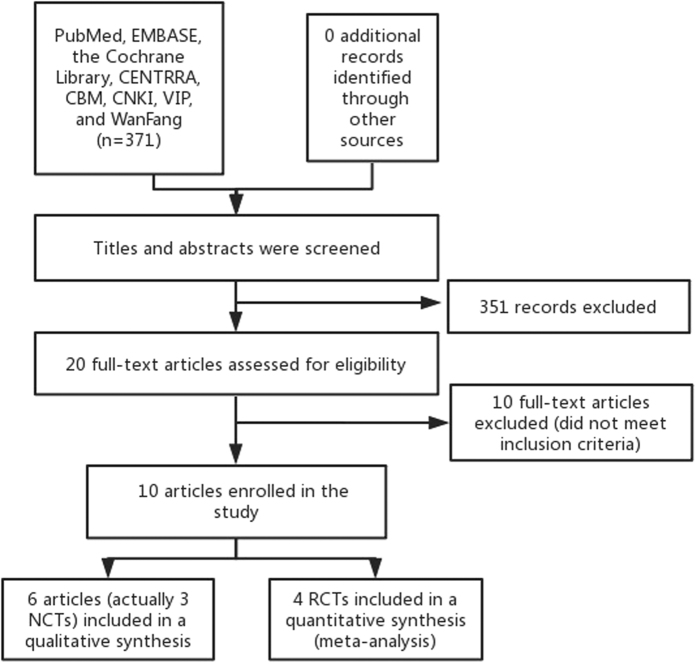
Flow diagram of study selection.

As is demonstrated in Fig. 1, a total of 371 articles were identified, 351 of which were determined to be irrelevant based on review of titles and abstracts. Thus, a total of 20 full-text articles were assessed for eligibility. Of these 20 articles, 10 were excluded because them didn’t meet the inclusion criteria, including four reviews, four animal experiments, two didn’t involved the outcomes. In total, 10 articles (actually 7 trials) fulfilled the inclusion criteria and were enrolled. A total of four RCTs^19^,^20^,^21^,^22^ were enrolled for meta-analysis. The remaining 6 articles were enrolled for systematic review, including five before-after studies^23^,^24^,^25^,^26^,^28^, 4 of which originated in one trial (clinical trial reg. no. NCT01392560)^23^,^24^,^25^,^28^. The others were retrospective reviews^27^, and all of the articles were reported in English. Of the 10 retrieved articles, 581 participants are represented. In total, the 4 RCTs represented 363 and 166 patients in the SGLT-2 inhibitors and comparator groups, respectively. The characteristics of the retrieved trials (including parameters of trials quality) and the recorded outcomes are reported in Table 1. The mean age, duration of DM, baseline HbA1c, body weight, and BMI of enrolled patients in the meta-analysis were 41 years, 21.28 years, 8.026%, 81.26 kg and 27.25 kg/m^2^, respectively.

### Quantitative analysis (Meta-analysis)

#### Efficacy of SGLT-2 inhibitors intervention

##### Effects on glycemic levels

**Figure 2:**
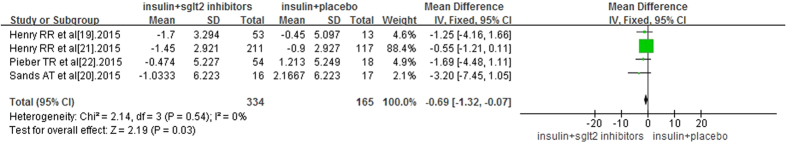
Forest plot for meta-analyses comparing SGLT-2 inhibitor with placebo in FPG. WMD = Weighted Mean Difference.

The four RCTs^19^,^20^,^21^,^22^ were summarized for meta-analysis. There was no significant heterogeneity between studies (P = 0.54, I^2^ = 0%). Compared with the placebo control, SGLT-2 inhibitors group significantly reduced FPG by 0.6 mmol/L [−1.30, −0.08] (P < 0. 05), as is shown in Fig. 2. The greatest reduction on FPG was observed with sotagliflozin 400 mg, which achieved a reduction of 3.2 mmol/l compared with placebo. A greater reduction was seen in the enrolled two before-after studies^23^,^24^,^25^,^26^,^28^, involved a combination of empagliflozin 25 mg and dapagliflozin 10 mg with insulin, and producted a significant reduction of FPG (by ~1.84 mmol/l) from baseline.

**Figure 3:**
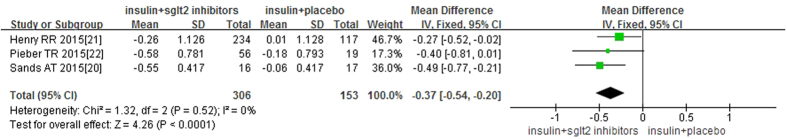
Forest plot for meta-analyses comparing SGLT-2 inhibitor with placebo in HbA1c. WMD = Weighted Mean Difference.

Three of the four RCTs^20^,^21^,^22^ assess the efficacy of SGLT-2 inhibitors on HbA1c. In these studies, the baseline HbA1C were all above 9.0%, and the use of SGLT-2 inhibitors produced an obvious improvement in HbA1C (WMD = −0.37%, 95% CI [−0.54, −0.20], P < 0.00001) (Fig. 3). Similarly, in three enrolled NCTs^23^,^24^,^25^,^26^,^27^,^28^, there was a reduction in HbA1c by 0.11% from baseline. Specifically, dapagliflozin in conjunction with insulin produced an obvious decline in HbA1c by 1.13%.

##### Effects on body weight

**Figure 4:**
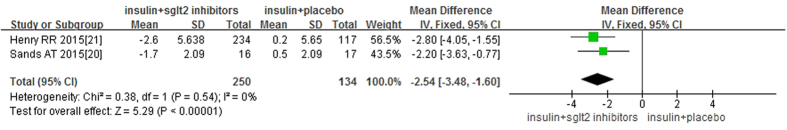
Forest plot for meta-analyses comparing SGLT-2 inhibitor with placebo in body weight. WMD = Weighted Mean Difference.

The effects of SGLT-2 inhibitors group versus placebo-control on body weight was explored in two RCTs^20^,^21^. In these studies, SGLT-2 inhibitors reduced body weight by 2.54 kg [3.48, 1.60] versus placebo-control group (Fig. 4). Similarly, two NCTs^23^,^24^,^25^,^26^,^28^, suggested a reduction in body weight by 2.18 kg, and reduction in body mass index (BMI) by 0.93 kg/m^2^ from baseline.

##### Effects on total daily insulin dose

**Figure 5:**
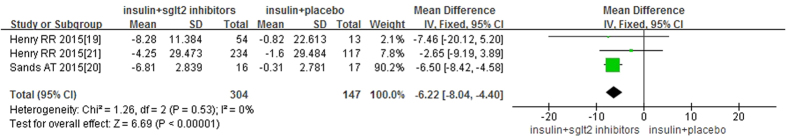
Forest plot for meta-analyses comparing SGLT-2 inhibitor with placebo in total daily insulin dose. WMD = Weighted Mean Difference.

All four RCTs^19^,^20^,^21^,^22^ showed that SGLT-2 inhibitors use with insulin could reduce TDI by 6.23 IU [8.05, 4.40] (P < 0. 00001) compared with placebo-control (Fig. 5), Pieber TR *et al*.^22^ corrected the data by body weight, and reported a decrease in insulin dose by 0.08 IU/kg (p < 0.05). A greater reduction was seen in two NCTs^23^,^24^,^25^,^27^,^28^, with a decrease of TDI by 7.85 IU from baseline.

### Safety of intervention

#### Total adverse events

**Figure 6:**
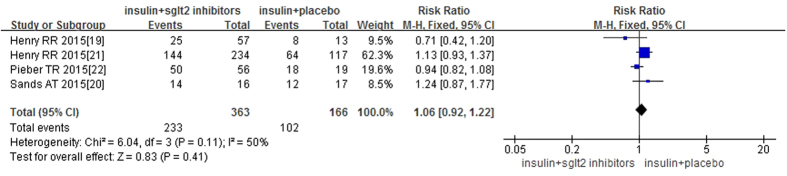
Forest plot for meta-analyses comparing SGLT-2 inhibitor with placebo in Total AEs. RR = related risk.

The use of SGLT-2 inhibitors associated with insulin versus placebo-control for the total incidence of AEs was studied in all four RCTs. There was no significant heterogeneity between the study (P = 0.11, I^2^ = 50%) and in using the fixed effects model, no statistically significant difference was seen for total risk of AEs for use of SGLT-2 inhibitors compared with placebo-controls, as shown in Fig. 6. Cherney *et al*.^23^ listed the AEs identified in their study, included volume depletion related AEs (such as thirst, polyuria, fatigue, etc.) and metabolism related AEs (such as hypoglycemia, diabetic ketoacidosis, etc.), they reported the highest incidence of AEs, with 40 (90%) cases of hypoglycemia and 34 cases (81%) of drug related AEs in empagliflozin group. However, the incidence of AEs with insulin monotherapy were not mentioned in this paper.

#### Hypoglycemia

**Figure 7:**
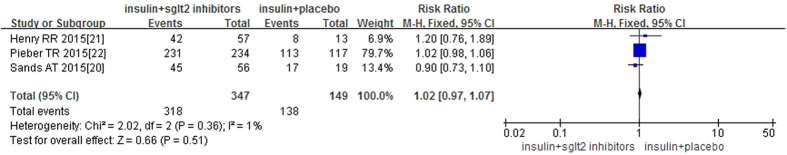
Forest plot for meta-analyses comparing SGLT-2 inhibitor with placebo in hypoglycemia. RR = related risk.

The incidence of hypoglycemic events was the outcome measure in three RCTs^19^,^21^,^22^. The incidence of severe hypoglycemic episodes (concurrent finger stick or plasma glucose <3.0 mmol/l or 54 mg/dl) was low, with 12 cases in SGLT-2 inhibitor group (n = 363) and 2 cases in placebo-control group (n = 166). A total of 4 cases were discontinued from study because of hypoglycemia, including 1 case which occurred in the dapagliflozin 10 mg group and 3 others which occurred in the canagliflozin 100 mg and 300 mg group. As is shown in Fig. 7, no statistically significant difference was noted for hypoglycemic episodes with SGLT-2 inhibitors versus placebo-controls.

#### Urinary tract or genital infection

**Figure 8:**
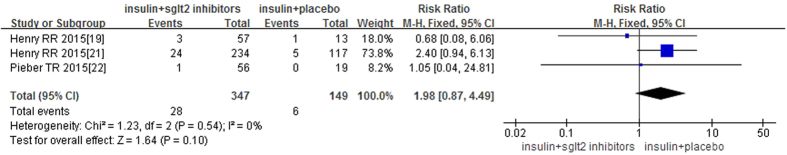
Forest plot for meta-analyses comparing SGLT-2 inhibitor with placebo in urinary tract or genital infection. RR = related risk.

Cherney and colleagues^23^ summarized the urinary tract or genital infection rates and reported 8 cases (19.05%) in the empagliflozin 25 mg combination group. The incidence of urinary tract or genital infection rates was similar among add-on group and placebo-control in the three RCTs^19^,^21^,^22^, as is shown in Fig. 8.

### Qualitative analysis

#### Other metabolic parameters

Two studies compared the effect of SGLT-2 inhibitor with placebo or baseline control on PPG^20^,^26^. Sands and colleagues^20^ observed effects on FPG assessed by Contin-uous glucose monitoring (CGM) during a three-hour period. The mean change from baseline at breakfast was −0.93 mmol/L and −0.23 mmol/L for sotagliflozin and the placebo control, respectively (P = 0.034). The changes from baseline at lunch and dinner were numerically lower for sotagliflozin than placebo, but did not reach a level of statistical significance. In a study by Tamez *et al*.^26^ which enrolled 12 patients with TIDM on insulin monotherapy, dapagliflozin 10 mg added to insulin improved PPG levels from baseline by −1.32 mmol/l (P = 0.08).

DM is inherently linked to the comorbidities of hypertension, plasma lipid metabolic disorder, and obesity, all of which are risk factors that may affect the incidence of cardiovascular events associated with the disease. Prior research has shown that cardiovascular disease is the main cause of death in adult patients with T1DM^29^. As a result there has been much interest as of late in evaluating the effects of anti-hyperglycemic agents on cardiovascular risk factors or cardiovascular events in DM. As the recently published EMPA-REG OUTCOME trial^31^ demonstrated, patients with T2DM treated with empagliflozin over three years experienced an overall 34% reduction in death from cardiovascular causes and hospitalization for heart failure, irrespective of whether or not they had heart failure when recruited into the study. Therefore, in addition to primary outcomes for the present study, we assessed total cholesterol, high-density lipoprotein cholesterol (HDL-C), low density lipoprotein cholesterol (LDL-C), triglycerides, blood pressure, and other metabolic parameters which were reported in retrieved articles, including one RCT^20^ and three NCTs^23^,^24^,^25^,^26^,^27^,^28^. A summary of these findings is provided in Table 2.

#### Diabetic ketoacidosis

Several cases of DKA were reported with the use of SGLT-2 inhibitors for treatment of both T1DM and T2DM^20^,^21^,^23^,^24^,^25^,^28^,^32^,^33^,^34^, while different from typically DKA which is associated with marked hyperglycemia and resultant dehydration, majority of the cases were occurred with a mild hyperglycemia or normoglycemia, the latter one, known as euglycemic or normoglycemic DKA (a BG concentration of <200 mg/dL), that raising valid questions regarding their clinical use and safety. DKA was observed by Henry *et al*.^20^, Sands *et al*.^21^ and Cherney *et al*.^23^,^24^,^25^,^28^ when SGLT-2 inhibitors were added to insulin for treatment of T1DM. There were a total of 16 DKA events. Henry *et al*.^20^ reported 17 cases (n = 234) of ketone related AEs, including 12 serious cases of DKA, while no patients in the placebo group (n = 117) had ketone related AEs. Sands *et al*.^19^ and Cherney *et al*.^23^,^24^,^25^,^28^ reported 2 cases of DKA, involving sotagliflozin 400 mg and empagliflozin 25 mg, respectively. However, all cases of DKA were considered in the context of precipitating factors (e.g., infection, pump failure, or cessation of insulin therapy)^35^.

## Discussion

The effective and safe use of oral hypoglycemic agents as adjunct therapy to insulin in T1DM has become a hot topic of interest in recent years. From reports of existing studies, there is an appreciation that any combination must be balanced against AEs, such as hypoglycemia, weight gain, and cardiovascular risk, among others. Aside from pramlintide, metformin was the most commonly used adjunct therapy to insulin in T1DM^11^, and studies of metformin indicated a significant reduction in weight, TDI, HbA1C, and putative effects on cardiovascular risk. However, HbA1C reduction was not statistically significant and could not be maintained to 6 months, and combination therapy may be associated with an increased incidence of gastrointestinal reactions^6^,^7^. As much of this continues to be under review, the final answer on the efficacy and safety of metformin in T1DM may come from the Reducing with Metformin Vascular Adverse Lesions in type 1 diabetes (REMOVAL) study (www.clinicaltrials.gov NCT01483560), which will be completed in 2016. Other oral hypoglycemic agents, such as alpha-glucosidase inhibitors and TZDs, have shown little or no promise in terms of glycemic control for patients with T1DM^5^. Alpha-glucosidase inhibitors provide a modest reduction in HbA1C and are associated with an increased risk of gastrointestinal reactions. The use of TZDs, however, pose a risk for development of AEs such as edema, weight gain, and possible worsening decline in endogenous insulin production^8^. Trials of GLP-1 analogs and DPP-4 inhibitors as insulin adjuncts in T1DM have also been conducted^9^,^10^, however, the effects remain to be seen.

SGLT-2 inhibitors, a new class of anti-diabetic agents with an insulin-independent mechanism, has proven efficacy in improved glycemic control, body weight, and blood pressure when used to treat T2DM. The effects on early glomerular hyperfiltration and urine protein excretion suggested a potential renal protective effect, and overall, the risks of hypoglycemia and urinary tract or genital infection were modest and well tolerated^13^,^14^,^15^,^16^. Several animal studies^17^,^18^ have been carried out using SGLT-2 inhibitors for treatment of T1DM. In a T1DM rat animal model, SGLT-2 inhibitors improved plasma glucose, increased urine glucose excretion (UGE) and protected remaining islet β-cell regeneration function. One proposed mechanism through which these outcomes are achieved is inhibition of oxidative stress induced by sugar toxicity. As a result, this has prompted further interest in the potential of SGLT-2 inhibitors for the treatment of T1DM in clinical trials.

The present study suggests that SGLT-2 inhibitors reduced FPG, HbA1c, body weight, and TDI compared with placebo or baseline, and that such therapy may improve cardiovascular risk factors such as blood pressure and plasma lipid profiles. As research regarding the use of SGLT-2 inhibitors for treatment of T1DM with renal insufficiency or cardiovascular disease is still lacking, efficacy in these instances should be further investigated. The four RCTs summarized for meta-analysis imply no increase in the total incidence of AEs, hypoglycemic events, or risk of urinary tract or genital infections in the combination therapy versus control group. This is in contrast from results of SGLT-2 use in T2DM which revealed an increased incidence of these outcomes. Potential reasons for discrepancies between the present study with regard to T1DM and prior studies of T2DM are (a) well-controlled glycemic levels with prior insulin therapy, thus decreasing UGE and the incidence of urinary tract or genital infection; (b) patients with T1DM were always young, who generally have the ability to resist infection; and (c) the relatively small sample size of most trials without specific mention of urinary tract or genital infections as a key finding.

Recently, several evidence suggests that SGLT-2 inhibitors may increase the incidence of DKA, especially in combination with insulin in T1DM or T2DM. A systematic review on SGLT2i in addition to insulin therapy for management of DM indicated 34 individual case reports of DKA, 25 cases involved patients with a diagnosis of T2DM, and 13 cases of DKA were reported in SGLT2i in addition to insulin therapy, including 5 cases occurred in T2DM and 8 cases in T1DM^36^, However, No RCTs on T2DM received SGLT2i in combination with insulin reported DKA events^37^. The possible mechanism by which SGLT2i might trigger DKA are as follows: a) SGLT2i induces lower blood glucose and thus reduces insulin secretion from pancreatic β-cells in turn, promotes the ketone bodies through activation of carnitine palmitoyltransferase-I (CPT-1) or β-oxidation in the liver. b) SGLT2i might stimulates the secretion of glucagon consequently to the decrease in insulin secretion, the direct effect of SGLT2i on pancreatic α-cells may also increase the production of glucagon, activate the lipase, promote adipose decompose and fatty acid oxidation, contribute to the overproduction of ketone bodies. c) It has been speculated that SGLT2i may increase the absorption of acetoacetate and inhibit the reabsorption of sodium in renal tubule, the latter may result in the change of electrochemical gradient, leading to the enhanced absorption of ketone body mediated by negative charge carrier of ketone^38^,^39^,^40^. The common precipitating factors included low β-cell reserve, decreased or discontinued insulin, stressful stimulations such as major surgery or illness, and other factors, for example, low carbohydrate diet, pancreatitis, dehydration, insulin delivery issue and alcohol was also found even though rare^36^. In May 2015, the Food and Drug Administration (FDA) warned that treatment with SGLT-2 inhibitors may increase the risk of DKA based on results of previous studies^41^. The American Association of Clinical Endocrinologists and American College of Endocrinology suggest that “in future T1D trials, lower SGLT-2 inhibitor doses should be considered and insulin dose should not routinely be reduced when SGLT-2 inhibitors are begun ……”^42^. While, a meta-analysis of RCTs revealed SGLT-2 inhibitor groups were not associated with a significantly higher risk of DKA compared with placebo or DPP-4 inhibitors in patients with T2DM^43^.

There is a limited number of RCTs available which studied the therapeutic effects of adjunct SGLT-2 inhibitors for treatment of T1DM. The retrieved RCTs included in the present study are all of high quality, however, there are still several potential limitations. One major limitation is the small sample size and short durations of studies included in this systematic review and meta-analysis. Most studies enrolled were less than 100 patients and were less than 12 weeks. Thus, a longer duration of observation with a larger powered study is needed to understand the long-term benefits and risks of SGLT-2 inhibitors in the treatment of T1DM. A second limitation is the use of varying types and dosages of SGLT-2 inhibitors among the studies, which inserts an obvious clinical heterogeneity between studies. Lastly, other potential confounding factors, for example, the effect of kidney function, the underlying heart and lung diseases, test method, quality of sample, survival time of red blood cell (RBC) and the effect of some other drugs were not analyzed. Yamout H *et al*.^44^ evaluated the efficacy and safety of canagliflozin in patients with type 2 diabetes and stage3 nephropathy, stated an reduction in HbA1c, body weight, blood pressure, and SGLT2i was generally well tolerated. At the same time, recent studies have also demonstrated an acute, dose-dependent reduction in estimated glomerular filtration (eGFR) rate by ≈5 mL·min^−1^·1.73 m^−2^ and ≈30% to 40% reduction in albuminuria in SGLT2i versus placebo or other anti-diabetic agents, independent of its effects on blood pressure or glucose control^45^,^46^, indicated a renoprotection role of SGLT2i in DM. Therefore, larger sample and various models study is needed, and needs to be updated at the end of a new clinical trials.

The effect of SGLT-2 inhibitors, with a unique mechanism of insulin-independent glucose disposal, represents a promising new therapy for T1DM. The available studies suggest that SGLT-2 inhibitors are an effective and safe insulin adjunct therapy, improving glycemic control, reducing body weight, TDI, and metabolic parameters such as blood pressure and plasma lipid profile. Taken together, the aforementioned suggests a potential benefit of cardiovascular and kidney protection, and the use of SGLT-2 inhibitors may play an important role in decreasing T1DM complications or alternatively delaying time to development of these complications, which is the topic directions for further evaluation on SGLT2i. Our current data shows that SGLT2i as an insulin adjunct in T1DM does not increase the incidence of total AEs, hypoglycemia, or genital and urinary infections, however, the risk of DKA should be carefully monitored. Additional prospective RCTs which are long-term, large sample sizes and various models will be required to definitively assess the adjunct use of SGLT-2 inhibitors in treatment of T1DM.

## Additional Information

**How to cite this article**: Chen, J. *et al*. The efficacy and safety of SGLT2 inhibitors for adjunctive treatment of type 1 diabetes: a systematic review and meta-analysis. *Sci. Rep.* **7**, 44128; doi: 10.1038/srep44128 (2017).

**Publisher's note:** Springer Nature remains neutral with regard to jurisdictional claims in published maps and institutional affiliations.

## Figures and Tables

**Table 1:** Baseline characteristics and quality assessment of trials included in the systematic review and meta-analysis (continued).

Study, Year	Participants	Study design	Interventions	HbA1 C (%)	FPG (mmol/l)	Body weight (kg)	Study duration (weeks)	Key findings	Quality assessment
Henry RR^19^	N:70 Age(years): 35.3	RCT	DAPA (1; 2.5; 5; 10 mg), PBO	8.46	8.58	75.63	2	24 h UGE; DAG; FP G; TDI	A
Sands AT^20^	N:33; Age(years): 39.58*	RCT	SOTA 400 mg; PBO	7.96	9.16	73.43*	4	HbA1C; FP G; body weight; TDI; AEs	A
Henry RR^21^	N:351; Age(years): 42.3	RCT	CANA (100 mg; 300 mg), PBO	7.9	NR	83.4	18	HbA1C; F PG; body weight; TDI; AEs	A
Pieber TR^22^	N:75; Age(years): 40.96	RCT	EMPA (2.5 mg; 10 mg; 25 mg), PBO	8.24	9.8	79.97	4	24 h UGE; MD G; HbA1C; FPG; body weight; TDI; AEs	A
Cherney DZ^23^,^24^,^25^,^28^	N:40; Age(years): 24.5	before-a fter study	EMPA 25 mg	8.0	9.0	72.6	8	Change in GFR; FPG; Hb A1C; body weight;	B
Tamez HE^26^	N:12; Age(years): 27.67	before-a fter study	DAPA 10 mg	9.18	9.81	78.33	24	FPG; PP G; HbA1C; weight and other metabol	B
Argento NB 2015^27^	N:27; Age(years): 51.1	Retros-p ective review	CANA 100 mg	7.65	NR	86.3	4	Weight; S BP; TDI; HbA1C; MDG; hy poglyce mia	B

CANA canagliflozin, DAPA dapagliflozin, EMPA empagliflozin, SOTA sotagliflozin, PBO placebo, postprandial plasma glucose PPG, TDI total daily insulin dose, HbA1C glycosylated hemoglobin, FPG fasting plasma glucose; age, FPG, HbA1C, body weight and duration of diabetes were reported in Mean, * for median, NR, not reported, A, low bias risk, B, unclear bias risk.

**Table 2:** Metabolic parameters assessed in qualitative analysis (all data were reported in Mean ± SD; NR, not reported).

	First author	Total cholesterol (mg/dl)	HDL cholesterol (mg/dl)	LDL cholesterol (mg/dl)	Triglycerides (mg/dl)	SBP (mmHg)	DBP (mmHg)
RCT	Sands AT 20	NR	NR	NR	NR	−1.0 ± 3.75	NR
NCT	Cherney DZ, Perkins BA 23,24,25,28	NR	NR	NR	NR	−2.1 ± 9.35	−0.95 ± 8.65
	Argento NB^27^	NR	NR	NR	NR	−8.9 ± 14.66	NR
	Tamez HE^26^	−100 ± 10.44	−2 ± 8.19	−17 ± 20.08	−24 ± 13.45	NR	NR
